# Bio-Waste Products of *Mangifera indica* L. Reduce Adipogenesis and Exert Antioxidant Effects on 3T3-L1 Cells

**DOI:** 10.3390/antiox11020363

**Published:** 2022-02-11

**Authors:** Giovanni Pratelli, Daniela Carlisi, Antonella D’Anneo, Antonella Maggio, Sonia Emanuele, Antonio Palumbo Piccionello, Michela Giuliano, Anna De Blasio, Giuseppe Calvaruso, Marianna Lauricella

**Affiliations:** 1 Department of Biomedicine, Neurosciences and Advanced Diagnostics (BIND), Institute of Biochemistry, University of Palermo, 90127 Palermo, Italy; giovanni.pratelli@unipa.it (G.P.); daniela.carlisi@unipa.it (D.C.); sonia.emanuele@unipa.it (S.E.); marianna.lauricella@unipa.it (M.L.); 2 Department of Biological, Chemical and Pharmaceutical Sciences and Technologies (STEBICEF), Laboratory of Biochemistry, University of Palermo, 90127 Palermo, Italy; michela.giuliano@unipa.it (M.G.); anna.deblasio@unipa.it (A.D.B.); giuseppe.calvaruso@unipa.it (G.C.); 3 Department of Biological, Chemical and Pharmaceutical Sciences and Technologies (STEBICEF), Section of Chemistry, University of Palermo, 90128 Palermo, Italy; antonella.maggio@unipa.it (A.M.); antonio.palumbopiccionello@unipa.it (A.P.P.)

**Keywords:** mango peel extracts, mango seed extracts, 3T3-L1 cells, adipogenesis, AMPK

## Abstract

Several studies highlighted the beneficial value of natural compounds in the prevention and treatment of obesity. Here, we investigated the anti-obesity effects of extracts of peel and seed of mango (*Mangifera indica* L.) cultivated in Sicily (Italy) in 3T3-L1 cells. Mango Peel (MPE) and Mango Seed (MSE) extracts at a 100 µg/mL concentration significantly reduced lipid accumulation and triacylglycerol contents during 3T3-L1 adipocyte differentiation without toxicity. HPLC-ESI-MS analysis showed that both the extracts contain some polyphenolic compounds that can account for the observed biological effects. The anti-adipogenic effect of MPE and MSE was the result of down-regulation of the key adipogenic transcription factor PPARγ and its downstream targets FABP4/aP2, GLUT4 and Adipsin, as well SREBP-1c, a transcription factor which promotes lipogenesis. In addition, both MPE and MSE significantly activated AMPK with the consequent inhibition of Acetyl-CoA-carboxylase (ACC) and up-regulated PPARα. The addition of compound C, a specific AMPK inhibitor, reduced the effects of MPE and MSE on AMPK and ACC phosphorylation, suggesting a role of AMPK in mediating MPE and MSE anti-lipogenic effects. Notably, MPE and MSE possess an elevated radical scavenging activity, as demonstrated by DPPH radical scavenging assay, and reduced ROS content produced during adipocyte differentiation. This last effect could be a consequence of the increase in the antioxidant factors Nrf2, MnSOD and HO-1. In conclusion, MPE and MSE possesses both anti-adipogenic and antioxidant potential, thus suggesting that the bio-waste products of mango are promising anti-obesity natural compounds.

## 1. Introduction

In recent years, the incidence of obesity significantly increased worldwide representing a health problem [1]. The expansion of white adipose tissue (WAT) which characterize obese patients results from a combination of factors, including overnutrition, unhealthy diet, reduced physical activity and genetic predisposition [2]. In a normal healthy person excess calories are stored as triacylglycerols (TGs) in WAT. When energy intake exceeds energy expenditure, this leads to a hypertrophic expansion of WAT which has been correlated with lipotoxicity and alteration of adipose tissue functionality [3,4]. Notably, a large body of literature reports that the hypertrophic WAT secretes adipokines with pro-inflammatory roles, which may directly interfere with insulin signaling and recruit macrophages that generate an inflamed state in the adipose tissue [5,6]. This status of low-grade of inflammation contributes to the development of different pathologies including type II diabetes, cardiovascular diseases and also certain forms of cancer [3,7,8,9].

The expansion of adipose tissue can be a consequence of two different events: accumulation of fat in existing adipocyte and differentiation of fibroblast such as pre-adipocytes in mature adipocyte by de novo adipogenesis. [10]. Therefore, the regulation of adipogenesis is significant for obesity prevention and treatment.

Several transcription factors regulating the expression of genes involved in adipocyte differentiation are activated during adipogenesis [11]. Among them, CCAAT enhancer binding protein alpha (C/EBPα) and peroxide proliferative activation receptor gamma (PPARγ) are master regulators [11]. They are involved in the stimulation of transcription factors and enzymes which promote lipid accumulation within adipocytes, such as sterol regulatory element-binding protein-1c (SREBP-1c), adipocyte fatty acid-binding protein 4 (FABP4), acetyl-CoA carboxylase (ACC) and fatty acid synthase (FAS) [12].

AMP-activated protein kinase (AMPK) is a nutrient sensor which is activated in response to cellular energy depletion [13]. To restore cellular ATP levels, AMPK stimulates energy-produced processes such as glycolysis, lipolysis and fatty acid oxidation, while inhibits energy-consuming process such as lipogenesis [13]. In adipocytes the activation of AMPK by phosphorylation of threonine 172 suppresses lipid biosynthesis. In particular AMPK phosphorylates and inactivates ACC, the enzyme involved in malonyl-CoA synthesis, as well as inhibits the expression of SREBP-1c, FABP4 and FAS [13]. This results in the attenuation of lipid accumulation in mature adipocytes. Recently, several studies highlighted that AMPK is involved in adipocyte differentiation. Particularly, AMPK activation inhibits adipogenesis by reducing the expression of C/EBPα and PPARγ in 3T3-L1 cells [14]. Therefore, AMPK activation could be beneficial to counteract adipogenesis.

Nowadays, several plants, due to the presence of bioactive compounds, have shown beneficial effects on the prevention and treatment of obesity by inhibiting adipogenesis, stimulating lipolysis and reducing chronic low-grade inflammation in adipocytes [15,16,17,18,19].

Mango (*Mangifera indica* L.) is a plant belonging to the *Anacardiaceae* family whose cultivation is widespread in tropical and subtropical areas of the world. In recent years, mango cultivation has also spread in different regions of Mediterranean area, including the South of Italy, which is characterized by a favorable subtropical climate and adapted soils for mango cultivation [20]. Mango fruit is highly appreciated all over the world not only for its aroma and pleasant taste, but because it is rich in active ingredients with an undisputed nutritional and nutraceutical value [20]. A vast literature highlights how different parts of the plant (leaves, flowers and bark) and of the fruit (peel, pulp and seed) contain phytochemicals capable of exerting anti-inflammatory, anti-oxidant and anti-tumoral effects [20,21,22,23]. Furthermore, mango pulp and leaf extracts have been reported to inhibit adipogenesis in mouse 3T3-L1 cells and improve plasma levels of pro-inflammatory cytokines in obese patients [24,25]. The nutraceutical properties of mango are linked to the presence of a wide range of polyphenols, including mangiferin, gallic acid, gallotannins, quercetin, isoquercetin, ellagic acid and β-glucogallin [20,22].

The edible part of the mango is only the pulp. Mango peel and seed are the main bio-wastes from mango processing, representing a consistent part of the fruit (35% to 60%). However, several studies report that these parts of the fruit also contain high levels of health-enhancing compounds with antioxidant and anti-tumoral activity [23,26,27]. Recently, it has been reported that mango peel extracts differently affect adipogenesis in 3T3-L1 cells in relation to the differences in phytochemical composition of mango cultivars [28,29]. However, the anti-adipogenic mechanism of mango peel needs to be clarified.

In the present study, we characterized bioactive compounds present in peel and seed of mango cultivated in Sicily (Italy) and examined the ability of these mango extracts in inhibiting adipogenesis in 3T3-L1 cells. This study provides evidence that both mango peel (MPE) and mango seed (MSE) extracts exert anti-adipogenic effects which seem to be mediated by downregulation of PPARγ and the activation of AMPK. In addition, our data highlight that MPE and MSE exert an anti-oxidant effect, counteracting ROS production during adipocyte differentiation.

## 2. Materials and Methods

### 2.1. Preparation of Mango Peel and Seed Extracts

Mango (*Mangifera Indica* L.) fruits grown in Sicily (Italy) were used in this study. Initially, the peel and seed were manually removed from the fruits, washed with distilled water, cut and lyophilized (Hetosicc Lyophilizer Heto CD 52-1). Next, the lyophilized products were powered using an electric blender, solubilized in ethanol-PBS 1:1 solution and kept overnight at 37 °C in constant agitation. Final concentration of both Mango Peel Extracts (MPE) and Mango Seed Extracts (MSE) was 75 mg/mL. Subsequently, the extracts were centrifuged at 120× *g* for 10 min and the supernatants were subjected to a subsequent centrifugation at 15–500× *g* for 10 min. MPE and MSE were stored in the dark at −20 °C until use. The working solutions of MPE and MSE were diluted to final concentration in the culture medium. The concentration of ethanol in the final solution did not exceed 0.06% of culture medium and was added as vehicle in control cells.

### 2.2. HPLC-ESI-MS Analysis

The lyophilized sample of Mango seed described above were solubilized as previously reported for mango peel [24]. The sample was subjected to ultrasound and vortex treatment, followed by filtration with 0.45 mm PTFE filters. The standard mangiferin calibration curve included 4 concentration points: 0.3, 0.45, 0.6 and 0.75 ppm. The standard gallic acid calibration curve included 4 concentration points: 1.5, 4.5, 7.5 and 15 ppm. All samples were analyzed in LC-MS/MS using the instrumentation: Q-Exactive LCq/Orbitrap MS, interfaced with UHPLC Ultimate 3000 RS in ESI (Electrospray Ionization). All experiments were performed in negative mode. The analyses were carried out using 2 different HPLC methods as previously reported [23]. MS total ion counts (TIC) was employed to monitor the eluate. Gallic acid and mangiferin standards were supplied by Sigma-Aldrich (St. Louis, MO, USA).

### 2.3. Cell Culture and Reagents

Mouse 3T3-L1 cell line was obtained from the American Type Culture Collection (ATCC). 3T3-L1 cells were cultured in complete DMEM (Euroclone, Pero, Italy) supplemented with 10% (*v*/*v*) heat-inactivated fetal bovine serum (FBS; Euroclone, Pero, Italy), 2 mM L-glutamine (BioWest, Nuaillé, France), 1% Non-Essential Amino Acids (BioWest, Nuaillé, France), 100 U/mL penicillin and 50 µg/mL streptomycin (Euroclone, Pero, Italy). The cells were maintained as monolayer in flasks of 75 cm^2^ at 37 °C in a 5% CO_2_ humidified incubator. When 3T3-L1 pre-adipocyte cells reached 80% of confluence, were detached from tissue culture flask using trypsin-EDTA (0.5 mg/mL trypsin and 0.2 mg/mL EDTA) and seeded in accordance to the experimental conditions. All reagents and compounds, except where differently reported, were purchased from Sigma-Aldrich (Milan, Italy).

### 2.4. Adipocyte Differentiation and Treatments

To obtain mature adipocytes, 3T3-L1 pre-adipocyte cells (undifferentiated cells, Undif. cells) were seeded at 0.2 × 10^5^/well in 24-well plate or 0.8 × 10^5^/well in 6-well plate and maintained in this state two days post-confluence. Then, confluent pre-adipocytes were incubated for 3 days in differentiation medium (MDI) (DMEM supplemented with 10% (*v*/*v*) heat-inactivated fetal bovine serum, 2 mM L-glutamine, 1% Non-Essential Amino Acids, 100 U/mL penicillin and 50 µg/mL streptomycin, containing the pro-differentiative agents 0.5 mM 3-isobutyl-1-methylxanthine (IBMX), 1 µM dexamethasone and 1 µg/mL insulin). Then, the culture medium was replaced and the cells were incubated for additional 5 days with maintenance medium (MM) (DMEM supplemented with 10% (*v*/*v*) heat-inactivated fetal bovine serum, 2 mM L-glutamine, 1% Non-Essential Amino Acids 100 U/mL penicillin and 50 µg/mL streptomycin containing 1 µg/mL insulin). To evaluate the effects of MPE and MSE, different doses (25, 50 and or 100 µg/mL) of each extract were added to MDI and MM until complete adipocytes differentiation. The culture medium and treatments were changed every two days and differentiation was completed at day 8. At this time the cells exhibited characteristic of mature adipocytes. Undifferentiated cells (Undif.) were grown in DMEM supplemented with 10% (*v*/*v*) heat-inactivated fetal bovine serum, 2 mM L-glutamine, 1% Non-Essential Amino Acids 100 U/mL penicillin and 50 µg/mL streptomycin. Adipocyte differentiation was evaluated based on the expression of adipogenic markers, Lipid droplets (LDs) formation and triglycerides accumulation. Control undifferentiated (Undif.) and differentiated adipocyte 3T3-L1 cells (Dif.) were treated with vehicle containing 0.06% ethanol. This concentration did not exert any toxic effects on the cells.

### 2.5. Cell Viability Assay

To evaluate cell viability, cells were treated with MTT 3-(4,5-dimethylthiazol-2-yl)-2,5-diphenyltetrazolium bromide which measures the activity of mitochondrial dehydrogenases as reported [30]. 3T3-L1 undifferentiated cells were plated in 96-well plate at a density of 8 × 10^3^/well. After 24 h the cells were exposed to different concentrations of MPE or MSE (25, 50, 75, 100, 150 and 200 µg/mL) for 8 days. MTT reagent (11 mg/mL in PBS, 20 μL) was added to each well and incubated for another 2 h at 37 °C. Then, the colored crystal of produced formazan was dissolved in 100 μL of lysis buffer (20% sodium dodecyl sulphate in 50% N,N-dimethylformamide, pH 4.0). The absorbance was measured by a microplate reader (OPSYS MR, Dynex Technologies, Chantilly, VA, USA) at 540 nm with a reference wavelength of 630 nm. Cell viability was measured as the percentage of the optical density (OD) values of treated cells compared with untreated cells as control.

### 2.6. Antioxidant Activity

Radical scavenging activity of MPE and MSE were determined by DPPH (1,1-diphenyl-2-picrylhydrazyl) radical. Different concentrations of MPE and MSE (25, 50, 75, 100 and 200 µg/mL) were added to ethanol DPPH solution (100 µM) in a final volume of 1 mL. Each concentration of extracts (A1) was incubated for 30 min in the dark at room temperature; then the loss of absorbance was measured at 517 nm spectrophotometrically. DPPH radicals have a maximum absorption at 517 nm, the peak disappears with reduction by an antioxidant compound. In the same way the negative control (A0) was prepared with ethanol DPPH solution, whereas a blank sample (A2) containing ethanol was used as reference. Radical scavenging activity (% of DPPH radical inhibition) was calculated using the following equation:

*Inhibition* (%) = 1 − (A1 − A2/A0 − A2) × 100

### 2.7. Western Blot Analysis

Protein levels were analyzed by Western blotting. After 8 days of adipocyte differentiation, cells were lysed as previously reported [31]. Protein concentration was evaluated by Bradford Protein Assay (Bio-Rad Laboratories S.r.l., Segrate, Milan, Italy). Next, 30 μg/sample of total proteins were resolved by sodium dodecyl sulfate (SDS)–polyacrylamide gel electrophoresis (PAGE) and blotted on a nitrocellulose membrane (Bio-Rad). For all immunodetection analyses were used specific primary antibodies against PPARγ (sc-7273), PPARα (sc-9000), FABP4/aP2 (sc-18661), Adipsin (sc-47683), GLUT4 (sc-53566), mMSOD (sc-133254), purchased from Santa Cruz Biotechnology (Santa Cruz, CA, USA); phospho-ACC (#07-303) purchased from EMD Millipore Corporation (Temecula, CA, USA); AMPKα (#2532); Thr172-Phosphorylated AMPKα (#2535) purchased from Cell Signaling (Danvers, MA, USA); SREBP-1c (#bs-1402R) purchased from BioSS (Dundee, United Kingdom); Nrf2 (NBP1-32822) purchased from Novus Biologicals (Bio-Techne SRL, Milan, Italy); HO-1, eme Oxygenase 1 (orb5455) purchased from Biorbyt Ltd. (Cambridge, United Kingdom). Subsequently, filters were incubated with HPR-conjugated secondary antibodies (Amersham, GE Healthcare Life Science, Milan, Italy), immunoreactive signals were detected using enhanced chemiluminescence (ECL) reagents (Cyanagen, Bologna, Italy) and the signals obtained were performed with ChemiDoc XRS (Bio-Rad, Hercules, CA, USA). The intensity of the protein bands was quantified using Quantity One 1-D Analysis software (Bio-Rad) and β-actin (A5060; Sigma-Aldrich) was used for bands normalization.

### 2.8. Oil Red O (ORO) Staining

The effect of MPE and MSE on adipogenesis was evaluated through Oil Red O staining (Sigma-Aldrich, St. Luois, MO, USA). Mature 3T3-L1 adipocytes, differentiated in a 24-well plate, were fixed with 10% formaldehyde for 1 h, washed with PBS and rinsed with 60% isopropanol for 5 min until completely dry. Next, the cells were stained with Oil Red O working solution (0.35 g in 100 mL isopropanol) for 10 min and then washed with dH_2_O several times. The pictures were obtained by using Leica DM-IRB microscope and images were acquired on a Leica DC300F digital camera using Leica IM50 software, as representative images of the experimental conditions. In addition, the pictures were analyzed in ImageJ, converted into high-contrast black and white images to visualize lipid droplets and scored as the percentage area per field [32]. Finally, Oil red O quantification was also performed by extracting the dye by 100% isopropanol for 10 min and the absorbance of the Oil Red O was measured at 490 nm; the percentage of the OD values of treated cells was compared with untreated cells as control.

### 2.9. Detection of Reactive Oxygen Species Generation

The cell-permeant 2′,7′-dichlorodihydrofluorescein diacetate (H_2_DCFDA) (Molecular Probe, Life Technologies, Eugene, OR, USA) dye was used to quantify the production of reactive oxygen species as previously reported [33]. For these experiments undifferentiated 3T3-L1 cells (0.2 × 10^5^/well) were seeded in 24-well plates and grown until to complete differentiation. At the end, the cells were washed with PBS and incubated with 10 μM H_2_DCFDA dye for 30 min in the dark in an incubator with 5% CO_2_ at 37 °C. Then, fluorochrome was removed, cells were washed in PBS and analyzed by fluorescence microscopy by using excitation and emission wavelengths that are appropriate for green fluorescence (FITC filter with λex = 485 nm and λem = 530 nm).

### 2.10. Triacylglycerol Accumulation Assay

3T3-L1 cells were differentiated for 8 days in the absence or presence of different concentrations of MPE or MSE (25, 50 or 100 μg/mL). Then, cells were lysed with 5% NP-40 and the triacylglycerol content of supernatants was quantified using a spectrophotometric commercial kit for Triglyceride determination (SENTINEL CH. SpA, Milan, Italy). Triacylglycerol concentrations were calculated based upon a standard curve made from triacylglycerol standard and normalized to total cellular protein content measured by Bradford assay as reported [34].

### 2.11. Statistical Analysis

Each experiment and all determinations were performed in triplicate. The data were represented as mean ± S.D. The statistical significance of the differences between single group and relative control was evaluated using a two-tailed Student’s *t*-test using Microsoft Excel. A *p* value < 0.05 was considered the threshold for statistical significance. Where not specified, the data is not significant with respect to the related control.

## 3. Results

Comprehensive characterization of the phenolic fraction of Sicilian mango seed was performed by HPLC-ESI-MS to compare the composition in water-soluble phenolic compounds in the two different parts of the fruit and the influence on the activity. As reported in the literature, gallic acid and its derivatives were the largest family found in mango flesh, peel and seed kernel samples [20]. HPLC-ESI-MS analysis of Mango seed extracts (MSE) evidenced the presence of 8 polar compounds in MSE (see Table 1). Quantification of mangiferin and gallic acid was performed with the calibration curves of their own standards. According to previous studies, the gallates, gallotannins and maclurin derivatives were quantified using the calibration curve of gallic acid.

Our previous studies demonstrated that in peel extracts (MPE) of Sicilian mango methyl digallate (487.15 mg/100 g), methyl gallate (225.87 mg/100 g), gallic acid (118.57 mg/100 g) and glucosyl gallate (108.14 mg/100 g) were the principal phenolic compounds [23]. Characterization of MSE evidenced higher concentration of polyphenols in MSE than MPE. In particular, methyl gallate was the major component (2520 mg/100 g) and methyl-digallate ester isomer (1210 mg/100 g) the second most abundant compound.

### 3.1. MPE and MSE Possess ROS Scavenger Activities

**Figure 1:**
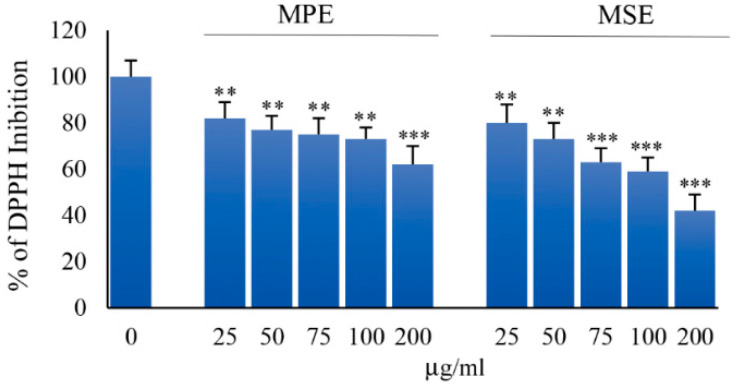
MPE and MSE possess radical scavenging activity. The antioxidant activity of MPE and MSE was evaluated by DPPH (1,1-diphenyl-2-picrylhydrazyl) radical scavenging assay. Different concentrations of MPE and MSE (25–200 µg/mL) were added on ethanol DPPH• solution and absorbance of each concentration was measured at 517 nm by spectrophotometer. The bar graphs represent the mean of three independent experiments ± SD. ** *p* < 0.01 and *** *p* < 0.001 with respect to the only vehicle control.

To ascertain the antioxidant activity of MPE and MSE we performed DPPH radical scavenging assay, as reported in Methods. Figure 1 shows the effect of different concentrations (25–200 μg/mL) of both the extracts. Our data demonstrated that both MPE and MSE possess an elevated radical scavenging activity. The inhibition of DPPH free radical exerted by mango extracts was dose-dependent and resulted higher in MSE than MPE, in accordance with the higher concentrations of polyphenols in MPE. In fact, at a concentration of 100 μg/mL, the DPPH inhibition exerted by MPE was only by 73%, respect to that of MSE that reached an inhibition value equal to 59%.

### 3.2. Effects of MPE and MSE on the Viability of 3T3-L1 Pre-Adipocytes

3T3-L1 cell line is one of the most widely used cell system for studying adipogenesis. In fact, these cells cultivated in the presence of a cocktail of pro-differentiative agents show the characteristics of mature adipocyte in metabolism and lipid accumulation [35].

**Figure 2:**
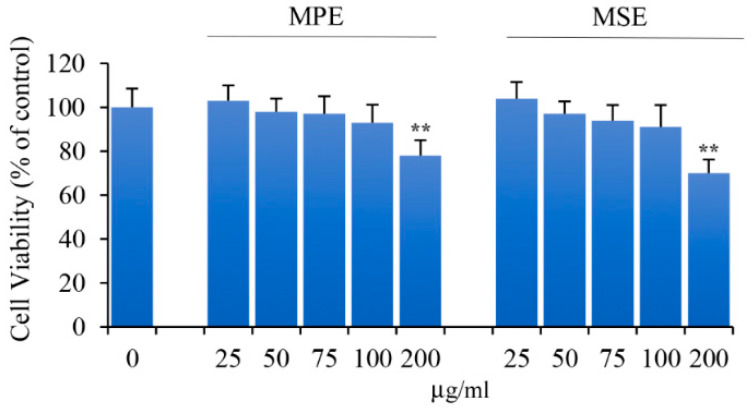
Effects of MPE and MSE on the viability of 3T3-L1 pre-adipocyte cells. 3T3-L1 cells (8 × 10^3^/200 μL of culture medium) were exposed to different doses (25–200 µg/mL) of MPE or MSE for 8 days. Then, the percentage of viable cells was assessed by MTT assay. The values reported are the mean ± SD of three independent experiments. ** *p* < 0.01 with respect to control cells treated with vehicle only.

Preliminary studies were performed to determine the non-toxic concentrations of MPE and MSE on 3T3-L1 pre-adipocytes undifferentiated (Undif.) cells. To this end, we evaluated the effect of different doses (25–200 µg/mL) of both the extracts on the viability of 3T3-L1 cells using the MTT assay. The results shown in Figure 2 indicate that 200 µg/mL of MPE or MSE slightly decreased the cell viability when compared to control cells after 8 days of treatment. Differently, the viability of 3T3-L1 cells was not affected by 100 μg/mL of both the extracts. 

### 3.3. MPE and MSE Reduced Lipid Content during 3T3-L1 Adipocyte Differentiation

**Figure 3:**
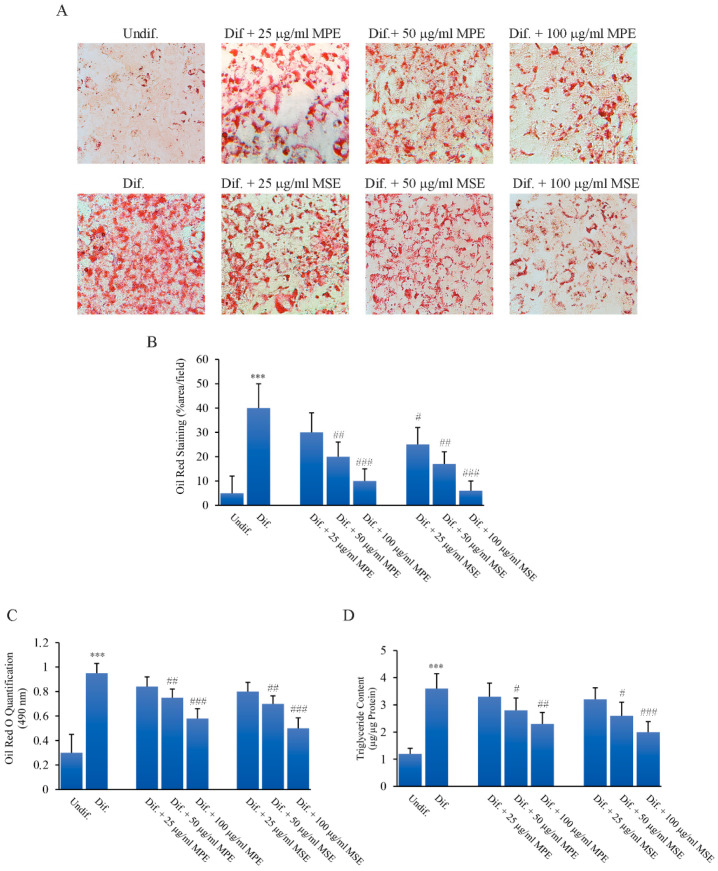
MPE and MSE reduce lipid content in 3T3-L1 adipocytes. 3T3-L1 cells were treated with pro-differentiative agents for 8 days in the presence or absence of different concentrations (25, 50 and 100 µg/mL) MPE or MSE, as reported in Methods. Representative photographs showing LDs reduction by Oil red O staining after MPE and MSE treatment (200× original magnification) (**A**). Lipid droplets (LDs) content was ascertained by analyzing the percentage area Red Oil stained by Image J (**B**) Quantitative Oil red O staining measured by spectrophotometer at 490 nm reading (**C**). Cellular TGs content quantified by spectrophotometer at 546 nm reading (**D**). The results are the mean of three independent experiments ± SD. *** *p* < 0.001 with respect to the undifferentiated cells (Undif.). # *p* < 0.05, ## *p* < 0.01 and ### *p* < 0.001 with respect to the differentiated untreated cells (Dif.).

Then, we evaluated the effects of MPE and MSE on the differentiation of 3T3-L1 cells into adipocytes. To this end, 3T3-L1 pre-adipocytes were differentiated for 8 days in the presence or absence of different doses (25, 50 and 100 μg/mL MPE or MSE), as reported in Methods. Results demonstrated that 3T3-L1 cells incubated with adipogenic medium appeared enlarged with the cytoplasm enriched of numerous droplets of various size, as observed by phase contrast microscopy (not shown). These morphological changes were less evident in 3T3-L1 cells differentiated in the presence of MPE or MSE. The addition of MPE or MSE also at the highest dose of 100 µg/mL did not exert toxic effects on the cells (not shown). Then, Oil Red O staining was applied to investigate the intracellular lipid accumulation as Lipid droplets (LDs). Microscopic examinations showed that differentiated 3T3-L1 cells increased Oil Red O staining compared with undifferentiated 3T3-L1 cells. Notably, MPE or MSE treatment markedly reduced the number and the size of LDs, compared to differentiated cells with a dose dependent effect (Figure 3A,B). Based on these results, 100 µg/mL of both MPE and MSE was chosen for further investigations.

LDs production was quantified by measuring the absorbance of the solubilized Oil Red O stained LDs at 490 nm. As shown in Figure 3C, both 100 µg/mL MPE and MSE reduced the absorbance of the stained cells by 31% and by 41%, respectively, compared to differentiated cells. These data suggest that MPE and MSE decreased the amount of lipids in 3T3-L1 adipocytes. Such a reduction in lipid accumulation was also sustained by measuring the triglycerides (TGs) content. The results showed that the intracellular TGs accumulation increased in differentiated cells by 200% relative to undifferentiated cells. Interestingly, we observed a significant dose-dependent decrease in TGs content. In particular, the addition of 100 µg/mL MPE or MSE significantly decreased the TGs content by 36% and 45%, respectively, compared to differentiated cells (Figure 3D).

### 3.4. MPE and MSE Reduced the Expression of Key Factors of Adipogenic Differentiation and Lipid Accumulation

**Figure 4:**
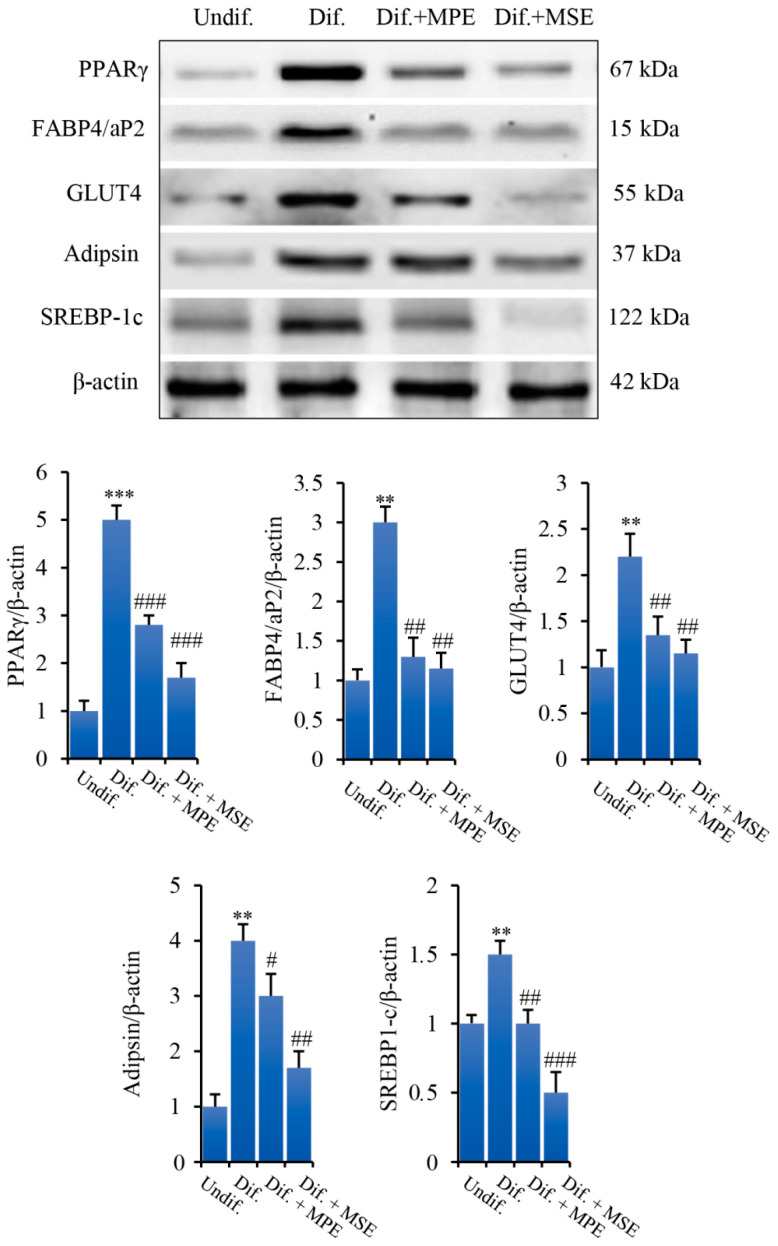
MPE and MSE down-regulate the expression of adipogenic differentiation and lipid accumulation markers in 3T3-L1 adipocytes. 3T3-L1 cells were treated with pro-differentiative agents for 8 days in the presence or absence of 100 µg/mL MPE or MSE, as reported in Methods. Then, cell lysates were analysed by Western blotting using specific primary antibodies directed against PPARγ, FABP4/aP2, GLUT4, Adipsin and SREBP1-c. Equal loading of proteins was verified by immunoblotting for β-actin and showed values were assigned in relation to undifferentiated cells (Undif.). The bar graphs represent the mean of three independent experiments ± SD. ** *p* < 0.01 and *** *p* < 0.001 with respect to undifferentiated 3T3-L1 cells (Undif). # *p* < 0.05, ## *p* < 0.01 and ### *p* < 0.001 with respect to the differentiated untreated 3T3-L1 cells (Dif.).

Peroxisome proliferator activated receptor γ (PPARγ) is a member of the nuclear hormone receptor superfamily. In the form of heterodimer with retinoid X receptor, PPARγ binds to the PPAR response element and modulate the expression of adipogenic and lipogenic genes [36]. PPARγ activation during adipocyte differentiation has been reported to be sufficient for adipogenesis in vitro and in vivo [37]. We observed that the level of PPARγ at differentiation day 8 markedly increased in differentiated 3T3-L1 cells respect to undifferentiated control cells (Figure 4). Notably, consistent with the decrease in lipid accumulation, lower levels of PPARγ were found in 3T3-L1 cells grown with adipogenic medium and treated with MPE or MSE (44% and 66%, respectively, in comparison with differentiated 3T3-L1 cells). Moreover, treatment with MPE or MSE resulted in a reduced expression of PPARγ downstream target genes. In fact, as evidenced in the same Figure 4, both MPE and MSE lowered in adipocytes the adipocyte fatty-acid binding protein (FABP4/aP2), a lipid-chaperone of adipose tissue whose level is greatly increased during the last stage of differentiation of adipocytes [38,39]; the glucose transporter 4 (GLUT4), a member of glucose transporter in adipose tissue involved in the insulin-stimulated glucose uptake [40]; and the Complement Factor D/Adipsin, an adipokine which promotes adipocyte differentiation and lipid accumulation [41].

We further examined the effects of mango extracts on the expression level of Sterol-regulatory element-binding protein-1c (SREBP-1c). SREBP-1c is a transcription factor upregulated under adipocyte differentiation which regulates the expression of genes involved in de novo lipogenesis and triglyceride synthesis, including ATP-citrate lyase (ACL), Acetil-Coa Carboxylase (ACC), Fatty acid synthase (FAS), Stearoyl-CoA desaturase (SCD1) and Glycerol-3-phosphate acyltransferase (GPAT) [42,43,44,45]. We demonstrated that the level of SREBP-1c increased by 50% in differentiated 3T3-L1 cells in comparison with undifferentiated cells. Interestingly, the addition MPE or MSE in the differentiation medium lowered the level of this factor (33% and 67%, respectively, in comparison with 3T3-L1 differentiated cells (Figure 4).

### 3.5. MPE and MSE Increase the Levels of Lipolytic Factors

**Figure 5:**
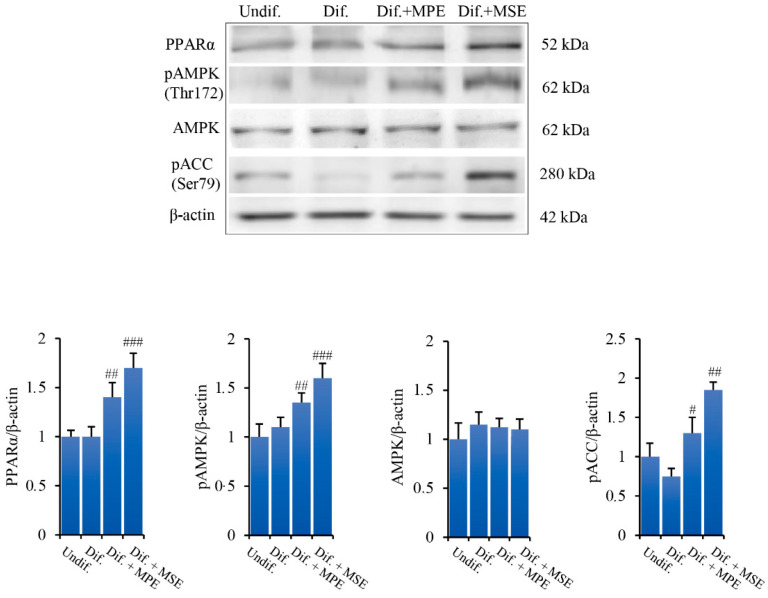
MPE and MSE up-regulate PPARα and activate AMPK in 3T3-L1 adipocytes. 3T3-L1 cells were treated with pro-differentiative agents for 8 days in the presence or absence of 100 µg/mL MPE or MSE, as reported in Methods. Then, cell lysates were analysed by Western blotting using specific primary antibodies directed against PPARα, AMPK, phospho-AMPK and phospho-ACC. Equal loading of proteins was verified by immunoblotting for β-actin and showed values were assigned in relation to undifferentiated 3T3-L1 cells (Undif). The bar graphs represent the mean of three independent experiments ± SD. # *p* < 0.05, ## *p* < 0.01 and ### *p* < 0.001 with respect to differentiated untreated cells (Dif.).

We further examined the ability of MPE and MSE to activate factors promoting lipolysis in adipocytes, such as PPARα and AMPK. Peroxisome proliferator-activated receptor-α (PPARα) is another member of PPAR family expressed mostly in tissues with high rates of fatty acid oxidation, such as the liver, brown fat and muscle [46]. It has been shown to up-regulate the expression of genes involved in fatty acid oxidation, particularly when co-activated by PPAR coactivator 1 (PGC-1) [47]. Our data, shown in Figure 5, demonstrated that the addition of MPE or MSE in the differentiation medium enhanced the expression level of PPARα (40% and 70%, respectively).

AMP-activated kinase (AMPK) is an energy sensor regulating glucose and lipid metabolism [13]. Upon activation in the phosphorylated form, AMPK reduces lipid synthesis by inhibiting the activity of the key enzymes of fatty acid de novo synthesis, such as ACC [13]. To investigate whether MPE and MSE are capable of activating AMPK, we examined by Western blotting analysis the levels of AMPK and its phosphorylated form. As shown in Figure 5, compared to differentiated 3T3-L1 cells, the presence of MPE or MSE in the differentiation medium enhanced the phosphorylation of AMPK by 23% and 45%, respectively. The results also showed that MPE or MSE did not significantly modify the basal levels of AMPK (Figure 5), thus supporting a role of mango extracts on AMPK activation. Concomitantly, the expression of the phosphorylated and inactive form of ACC increased following MPE and MSE treatment by 44% and 110%, respectively.

**Figure 6:**
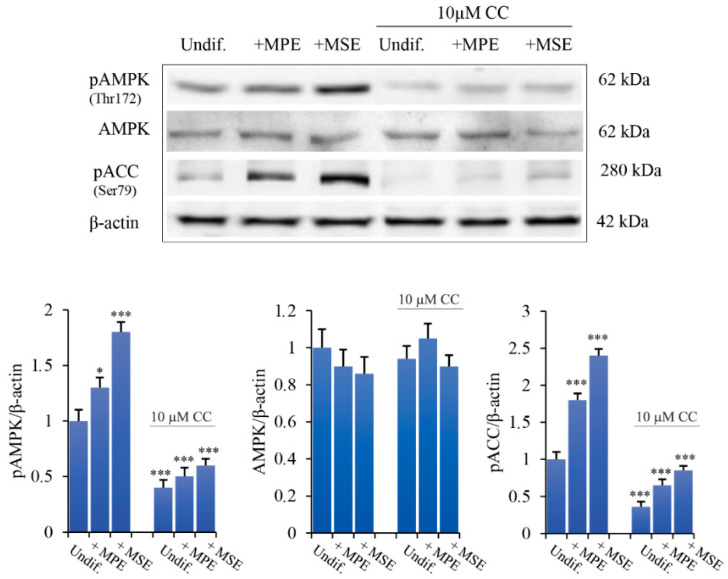
Compound C counteracts the effects of MPE and MSE on AMPK activation and ACC phosphorylation. Confluent 3T3-L1 cells were pre-treated for 4 h with 10 μM AMPK inhibitor, compound C (CC), and for another 24 h in the presence or absence of 100 µg/mL MPE or MSE. Cell extracts were then prepared and immunoblotted with antibodies to AMPK, phospho-AMPK, or phospho-ACC. Equal loading of proteins was verified by immunoblotting for β-actin and showed values were assigned in relation to undifferentiated 3T3-L1 cells (Undif). The bar graphs represent the mean of three independent experiments ± SD. * *p* < 0.05 and *** *p* < 0.001 with respect to the undifferentiated untreated 3T3-L1 cells.

To ascertain whether mango extracts act via AMPK, confluent 3T3-L1 pre-adipocytes were pre-treated for 4 h with Compound C (10 µM, CC), a highly selective inhibitor of AMPK [48]. Then, the cells were treated for 24 h in the presence of 100 μg/mL MPE or MSE. Notably, CC counteracted the effects of MSE and MPE on AMPK and ACC phosphorylation, while did not affect the basal level of AMPK (Figure 6). These results demonstrate that MPE and MSE are capable of activating AMPK with consequent inhibition of ACC, suggesting a role of this factor in reducing lipogenesis. 

### 3.6. MPE and MSE Reduce ROS Production in Adipocytes

**Figure 7:**
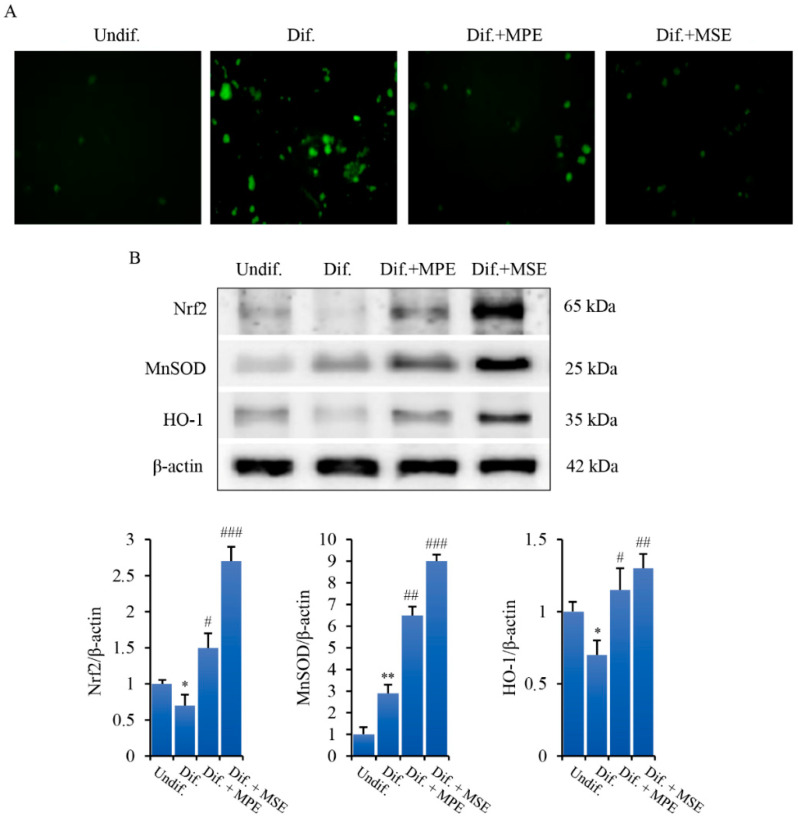
MPE and MSE exert anti-oxidant effects in 3T3-L1 adipocytes. 3T3-L1 cells were treated with pro-differentiative agents for 8 days in the presence or absence of 100 µg/mL MPE or MSE, as reported in Methods. (**A**) Intracellular ROS were detected using the redox-sensitive fluorochrome H_2_-DCFDA. After differentiation, the medium was replaced with 10 µM H2DCFDA solution and the incubation was protracted for 30 min at 37 °C. The oxidation of the fluorochrome generates green fluorescence, which was visualized by a Leica microscope equipped with a DC300F camera using a FITC filter. Representative micrographs of fluorescence microscopy were taken at 200× magnification. (**B**). Western blotting analysis of Nrf2, MnSOD and HO-1 in 3T3-L1 cells differentiated without or with 100 µg/mL MPE or MSE. Equal loading of proteins was verified by immunoblotting for β-actin and showed values were assigned in relation to undifferentiated cells (Undif.). The bar graphs represent the mean of three independent experiments ± SD. * *p* < 0.05, ** *p* < 0.01 with respect to the undifferentiated 3T3-L1 cells, # *p* < 0.05, ## *p* < 0.01 and ### *p* < 0.001 with respect to the differentiated untreated 3T3-L1 cells (Dif.).

It has been reported that ROS generation favours adipogenesis by regulating clonal expansion during adipocyte differentiation [46]. To ascertain ROS involvement in our model system, we performed fluorescence microscopy analysis by employing the fluorochrome H_2_DCFDA, a dye employed as a general indicator of intracellular ROS levels. As evidenced in Figure 7A, differentiated 3T3-L1 cells exhibited a pronounced green fluorescence detectable using fluorescence microscopy which is indicative of intracellular ROS production. Such an effect was consistently attenuated by 100 µg/mL MPE and counteracted by 100 µg/mL MSE.

In the next phase of our experiments, studies were carried out to ascertain the mechanism responsible for the anti-oxidant effect of MPE and MSE. In this regard, the analyses were focused on Nrf2, one of the major transcription factors that promotes cellular defence against oxidative stress [42]. Our data show that Nrf2 level is enhanced in adipocytes differentiated in the presence of MPE or MSE. In fact, in comparison with differentiated control cells the level of Nrf2 protein increases with MPE or MSE by about 110% and 280%, respectively (Figure 7B).

Superoxide dismutase (MnSOD) and Heme oxygenase (HO-1) are ROS scavenger enzymes transcriptionally regulated by Nrf2 [49]. We demonstrated that the levels of these proteins markedly increased after treatment with MPE or MSE (Figure 7B). In particular, the increase in the presence of 100 μg/mL MPE or MSE is estimated to be 120% and 210% for MnSOD and 64% and 86% for HO-1. Taken together, these results seem to indicate that the activation of Nrf2 plays a role in the antioxidant behaviour of MPE and MSE.

## 4. Discussion

This paper aimed at investigating the anti-adipogenic effect of peel and seed from mango cultivated in Sicily in 3T3-L1 adipocytes. Sicily is a region of the Southern Italy characterized by a favorable subtropical climate and adapted soils that favour mango cultivation conferring particular properties to the orchards [20]. 

It is worthwhile to mention that, although the chemical investigation of different phytocomponents of mango fruit has been already published by other researchers, in this paper we focused on cultivars of mango grown in the Sicilian rural areas to provide an analysis of their specific composition. Indeed, many different factors can affect the plant phytochemical profile including environmental factors, mango variety, course of fruit ripeness [50]. Such an aspect was also reported by Ajila et al. [51] providing evidence that MPE polyphenolic content strongly depends on fruit maturity stage at the time of harvest, favorable climatic conditions as well as growing location [52]. Interestingly, the characterization of the polyphenolic profile of MPE and MSE using HPLC/MS provided evidence that both these fractions of mango are rich in polyphenols. In particular, as demonstrated in a previous study [23], methyl digallate, methyl gallate, gallic acid and glucosyl gallate were the principal phenolic compounds in MPE. The polyphenols profile of MSE is similar to MPE, with methyl digallate and methyl gallate representing the main components. Notably, these polyphenols are at higher concentration in MSE than MPE. Gallic acid and its derivatives methyl gallate, methyl digallate and glucosyl gallate are plant secondary polyphenolic metabolites which possess strong anti-oxidant effects, due to their ability to act as ROS scavenger [53]. Interestingly, gallic acid exerts protective effects against obesity-related inflammation by reducing adipocyte size and the inflammation markers, as IL-6, NOS and COX2 [54]. Notably, methy gallate has been shown to exert anti-adipogenic effects in 3T3-L1 cells and human subcutaneous adipocytes, by reducing triglyceride content and down-regulating adipocyte differentiation markers as C/EBPα, PPARγ and FABP4 [55].

Significantly, our results provided evidence that Sicilian MPE and MSE have strong effects on reducing adipogenesis and lipid accumulation in 3T3-L1 adipocytes. Adipogenesis resulting from differentiation of pre-adipocytes into adipocytes leads to intracellular lipid accumulation [10,56]. Interestingly, our data demonstrated that accumulation of Lipid droplets (LDs) and triacylglicerols (TGs) induced by adipocyte differentiation was largely reduced by treatment of MPE and MSE compared with untreated differentiated cells, thus indicating their promising role as anti-obesity agents by inhibition of lipid accumulation. Notably, the highest dose of MPE and MSE (100 μg/mL) which exerts anti-adipogenic effects did not show toxic effects on both pre-adipocyte and differentiated 3T3-L1 cells. Therefore, inhibition of TGs accumulation by mango extracts seems to be related to reduction in adipogenesis without cytotoxicity.

To understand the molecular mechanism underlying the anti-adipogenic effect of MPE and MSE, we then focused our attention on some key factors involved in both adipocyte differentiation and lipid metabolism. Adipogenesis is a complex process which is tightly regulated by sequential activation of various transcriptional factors [11]. PPARγ is a member of the nuclear hormone receptor family expressed in adipose tissue. Its level increases at an early stage of this differentiation process to stimulate the expression of many adipocyte-specific genes which control fatty acid metabolism [36]. Induction of PPARγ has been shown to be necessary for adipogenesis both in vitro and in vivo and in many cases sufficient to convert non-adipose cells to adipocyte-like cells [37,57]. Notably, both MPE and MSE significantly reduced the expression level of PPARγ consistent with the decrease in lipid accumulation compared with untreated adipocytes. In addition the presence of mango extracts during adipogenic differentiation led to a reduction in the levels of FABP4 and GLUT4, two markers of late adipogenesis which are transcriptionally regulated by PPARγ [58]. In addition, we found that MPE and MSE significantly lowered SREBP-1c, a member of the basic helix–loop–helix-leucine zipper family of transcription factors, which has been shown to have an important role in adipogenesis [42]. Taken together, our data suggest that mango extracts could counteract adipogenesis by down-regulating the expression of PPARγ and SREBP-1c.

In addition to downregulate transcription factors involved in the stimulation of adipogenesis, our results demonstrated that MPE and MSE also up-regulated factors promoting catabolic process in adipocytes. PPARα is another member of PPAR family mainly expressed in liver and muscle cells [46] which promotes fatty acid oxidation [47]. Its level is low in white adipose tissue suggesting a limited role for this isotype during adipogenesis [46]. Of note however, pharmacologic PPARα activators reduced adiposity in mouse models of obesity [59]. Our data demonstrated that MPE and MSE up-regulated PPARα levels, thus supporting a role of this transcription factor in reducing lipid accumulation in 3T3-L1 adipocytes.

Interestingly, we also demonstrate that MPE and MSE increased the phosphorylated and active form of AMPK. This result is in line with the observation that many polyphenols derived from plants, such as resveratrol, quercetin, genistein and epigallocatechin gallate, are able to activate AMPK [60]. This activation seems to be mediated by the increase in AMP levels as a consequence of inhibition of mitochondrial ATP production [60]. Notably, AMPK activation could favor the inhibition of adipogenesis induced by MPE and MSE. This conclusion is in accordance with observation that AMPK negatively regulates white adipocyte differentiation [61]. To this end, 5-Aminoimidazole-4-carboxamide-1-β-D-ribofuranoside (AICAR), an activator of AMPK, led to the inhibition of differentiation in 3T3-L1 pre-adipocytes and such an effect was accompanied by decreased PPARγ and C/EBPα [62]. Moreover, the anti-adipogenic effects of several natural compounds seem to be mediated by AMPK activation [63,64,65].

In concomitance with activation of AMPK, we observed an increase in the phosphorylated and inactive form of ACC. ACC is the key rate-limiting enzyme in the first stage of fatty acid synthesis and it is inactivated via phosphorylation by AMPK [66]. Notably, we observed that the addition of compound C, a specific AMPK inhibitor, counteracted the effects of MPE and MSE on phosphorylation of both AMPK and ACC, thus demonstrating the ability of mango extracts to activate AMPK and suggesting a role of this factor in inhibiting lipogenesis and adipogenesis in 3T3-L1 cells. 

Reactive oxygen species (ROS) generation has been observed during adipogenesis and seems to promote adipocyte differentiation [67]. In particular, it has been shown that ROS increased PPARγ in early pre-adipocyte differentiation and promotes mitotic clonal expansion of pre-adipocytes [68].

In addition, ROS production has been correlated with enhanced mitochondrial biogenesis and metabolism during adipogenesis [69]. To support this conclusion it has been shown that mitochondrial-targeted antioxidants inhibited adipocyte differentiation, while the addition of hydrogen peroxide restored it [70]. Notably, our results showed that both MPE and MSE are capable of counteracting ROS production during 3T3-L1 adipocyte differentiation. This is in line with the observation that mango extracts possess a strong scavenger activity, as demonstrated by the ability of MPE and MSE to significantly inhibit DPPH activity.

Nrf2 is the master regulator of the cellular antioxidant response, regulating the expression of a battery of genes encoding for antioxidant and detoxifying factors [71]. Under normal condition, kelch-like ECH-associated protein 1 (Keap1) binds Nrf2 in an inactive complex, leading to its ubiquitin-proteasomal degradation. Under oxidative stress condition, ROS promote oxidation of Keap1 in a critical cysteine residue, promoting its dissociation from Nrf2 [63]. Thus, Nrf2 traslocates into the nucleus where up-regulate a panel of antioxidant genes, including superoxide dismutase (SOD) and heme oxygenase (HO-1). In accordance with this observation, we demonstrated that during adipocyte differentiation MPE or MSE upregulated Nrf2 and its targets HO-1 and MnSOD. Thus, the induction of these anti-oxidant enzymes could explain MPE- and MSE-induced ROS reduction in adipocytes. Several reports suggest that different natural compounds exert anti-oxidant effects by activating Nrf2 through different mechanisms including interaction with cysteine residues on Keap1, disruption of Nrf2/Keap1 interaction or Nrf2 phosphorylation [72]. Notably, Nrf2 and AMPK has been shown to be functionally connected and collaborate to reduce oxidative stress. Induction of AMPK by natural compounds [73] or chemical activators [74] leads in turn to activation via phosphorylation of Nrf2. This has been correlated with a reduction in inflammation in several cell types, as adipocytes, macrophages and pancreatic cells [74,75]. Thus, MPE and MSE could explain antioxidant effects by promoting activation of both AMPK and Nrf2 in 3T3-L1 adipocytes.

## 5. Conclusions

**Figure 8:**
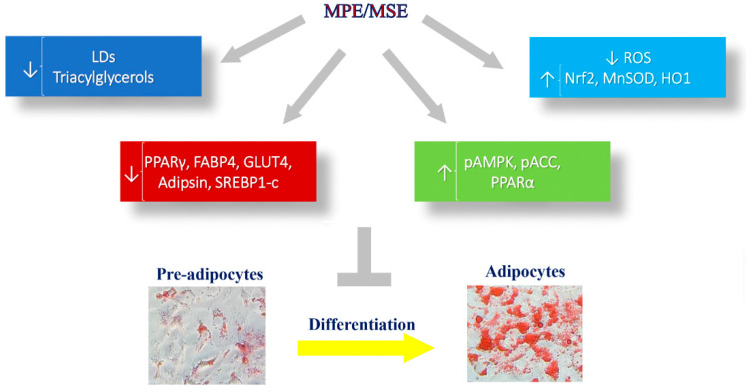
The addition of MPE and MSE to the differentiation medium reduced 3T3-L1 adipocytes differentiation, as demonstrated by the loss of LDs and TGs. The anti-adipogenic and anti-lipogenic effects of MPE and MSE seem to be correlated with the ability of mango extracts to reduce PPARγ and SREBP-1c and their targets, as well as to increase AMPK and PPARα. MPE and MSE also reduce ROS content produced during adipocyte differentiation, by activating Nrf2 and its downstream targets.

In conclusion, our results demonstrated that MPE and MSE reduce adipogenesis in 3T3-L1 cells by inhibiting lipid accumulation, down-regulating the expression of adipogenic genes and inhibiting lipogenic enzyme activity. Our data also suggested that AMPK activation contributed to these effects. Furthermore, we demonstrated that MPE and MSE possess strong antioxidant effects which could be mediated by activation of Nrf2-signalling pathway and contribute to reduce adipogenesis (Figure 8). HPLC/MS characterization of the polyphenolic profile of MPE and MSE provided evidence that both mango extracts are a good source of polyphenols which can explain the anti-oxidant and anti-adipogenic effect of these mango fractions. Studies are in progress to characterize the effects of single compounds present in the extracts. Mango peel powder has been incorporated in food as macaroni and biscuits by improving their nutraceutical proprieties [51]. Thus, bio-waste products from mango, such as peel and seed, could be used as a potential supplement to improve the nutritional value of food with health benefits in the prevention of overweight and obesity.

## Figures and Tables

**Table 1:** Composition of mango seed extract (MSE) HPLC-ESI-MS analysis of Mango seed extracts (MSE) evidenced the presence of 8 polar compounds in MSE. Quantification of mangiferin and gallic acid was performed with the calibration curves of their own standards.

	Compound	RT (min)	ESI [M − H] (m/z)		Molecular Formula	ppm	mg/100 g
			**Teor.**	**Exp.**			
1	Disaccaride	1.5	341.1089 [M − H]^−^	341.1089 [M − H]^−^	C_12_H_22_O_11_	trace	trace
2	Quinic acid	1.5	191.0561 [M − H]^−^	191.0561 [M − H]^−^	C_7_H_12_O_6_	trace	trace
3	Glucosyl gallate	4.6	331.0671 [M − H]^−^	331.06707 [M − H]^−^	C_13_H_16_O_10_	2.80	280
4	Gallic acid	5.8	169.0142 [M − H]^−^	169.01425 [M − H]^−^	C_7_H_6_O_5_	1.3	130
5	Methylgallate	15.3	183.0299 [M − H]^−^	183.02990 [M − H]^−^	C_8_H_8_O_5_	25.2	2520
6	Mangiferin	20.9	421.0776 [M − H]^−^	421.07763 [M − H]^−^	C_19_H_18_O_11_	0.5	50
7	Methyl-digallate ester isomer	22.6		335.04086 [M-H]^-^		12.1	1210
8	Maclurin tri-O-galloyl-glucoside	29.0	879.1262 [M − H]^−^	879.11651 [M − H]^−^	C_40_H_32_O_23_	trace	trace

## Data Availability

The data presented in this study are available in this manuscript.

## Abbreviations

ACC	acetyl-CoA carboxylase
ACL	ATP-citrate lyase
AMPK	AMP-activated protein kinase
C/EBPα	CCAAT enhancer binding protein alpha
Compound C	CC
DPPH	1,1-diphenyl-2-picrylhydrazyl radical
FABP4/aP2	adipocyte fatty acid-binding protein 4/adipocyte protein 2
FAS	fatty acid synthase
FBS	fetal calf serum
GLUT4	glucose transporter 4
GPAT	glycerol-3-phosphate acyltransferase
H_2_DCFDA	2′,7′-dichlorodihydrofluorescein diacetate
HO-1	heme oxygenase
HPLC-ESI-MS	high-performance liquid chromatography/electrospray ionization tandem *mass spectrometry*
IBMX	3-isobutyl-1-methylxanthine
Keap1	kelch-like ECH-associated protein 1
LDs	lipid droplets
MM	maintenance medium
MnSOD	manganese superoxide dismutase
MDI	differentiation medium
MPE	mango peel extract
MSE	mango seed extract
MTT	3-(4,5-dimethylthiazol-2-yl)-2,5-diphenyltetrazolium bromide
Nrf2	NF-E2–related factor 2
ORO	Oil Red O
PGC-1	PPAR coactivator 1
PPARα	peroxide proliferative activation receptor alfa
PPARγ	peroxide proliferative activation receptor gamma
ROS	Reactive oxygen species.
SREBP-1c	sterol regulatory element-binding protein-1c
SCD1	stearoyl-CoA desaturase
TGs	triacylglycerols
WAT	white adipose tissue
